# Perceptions of the Coach–Athlete Relationship Predict the Attainment of Mastery Achievement Goals Six Months Later: A Two-Wave Longitudinal Study among F. A. Premier League Academy Soccer Players

**DOI:** 10.3389/fpsyg.2017.00684

**Published:** 2017-05-18

**Authors:** Adam R. Nicholls, Keith Earle, Fiona Earle, Daniel J. Madigan

**Affiliations:** ^1^School of Life Sciences, University of HullHull, United Kingdom; ^2^School of Sport, York St John UniversityYork, United Kingdom

**Keywords:** coaching, goal attainment, performance, mastery-approach goals, relationships

## Abstract

All football teams that compete within the F. A. Premier League possess an academy, whose objective is to produce more and better home-grown players that are capable of playing professionally. These young players spend a large amount of time with their coach, but little is known about player’s perception of the coach–athlete relationship within F. A. Premier League Academies. The objectives of this study were to examine whether perceptions of the coach–athlete relationship changed over six months and if the coach–athlete relationship predicted self-reported goal achievement among F. A. Premier League academy players. This study included cross-sectional (*n* = 104) and longitudinal (*n* = 52) assessments, in which academy soccer players completed a measure of the coach–athlete relationship and goal achievement across either one or two time periods. The cross-sectional data were subjected to bivariate correlations, whereas the longitudinal data were analyzed using multiple regressions. Perceptions of the coach–athlete relationship remained stable over time. The coach–athlete relationship predicted the achievement of mastery goals six months later. Enhancing the quality of the coach–athlete relationship among elite adolescent athletes appears to be a suitable way of maximizing mastery achievement goals, particularly among developmental athletes who participate in team sports.

In response to concerns about the low number and quality of young players produced by soccer academies within England and Wales, the academy managers from Premier League clubs met up to discuss plans to modernize the structure of soccer academies, with a view to producing more and better homegrown players. This meeting and subsequent consultations resulted in the development of the Elite Player Performance Plan (EPPP), which details the processes and procedures necessary for academies to produce more and better homegrown players. This revolved around producing world leading coaching, creating tactically and technically excellent players who are independent decisions makers, and developing educationally rounded people (Football Association Premier League, 2011). As such, all academies are working toward achieving these goals and there is a clear structure across professional clubs.

F. A. Premier League academies are for players talented players aged from Under-9s through to Under-21s age groups. Academies are divided into three phases: foundation (Under-9 to Under-11), youth development (Under-12 to Under-16), and professional development (Under-17 to Under-21). Academy players in the foundation and youth development phases combine playing in the academy with their school responsibilities. Conversely, players in the professional development phase are full-time players, although there are educational commitments for Under-18 players. Even though being an F. A. Premier League academy player is very prestigious, these players are also expected to commit many hours to training and playing matches, with a typical season running from mid-July to mid-June (Morley et al., 2014). As such, over the course of a season, academy players will spend many hours working with their coach to improve their technique and tactical understanding of football (Reeves et al., 2011a). Indeed, players in the foundation age groups spend 8 h a week with their coach on the pitch and an additional 2 h each week with sport science support staff (e.g., strength and conditioning coaches, nutritionists, sport psychologists, and performance analysts). Players in the development groups spend 8 h with their coach on the pitch training, and a further 4 h with sport science support staff. The professional development phase consists of two groups. The Under-18 players are classified as scholarship players and spend 12 h with their coach on the pitch, 2 h in the classroom with their coach, 10 h with sport science support staff, and 6 h in education. Finally, the Under-21 players are with their coach on the pitch for 12 h per week and additional 2 h in the classroom. They accumulate 11 h per week with sport science support staff, but have no formal education. There are potentially other times in which the players and athletes may develop their relationship, such as when they spend time traveling to matches or if they spend time away on tour or at tournaments.

Jowett (2007) defined the coach–athlete relationship as all situations in which a coach’s and athlete’s feelings, thoughts, and or behaviors are inter-related. The quality of the relationship between an athlete and his or her coach is therefore very important. The coach–athlete relationship may impact upon a player’s happiness (Lafrenière et al., 2011), coping (Nicholls et al., 2016b), the generation of challenge or threat states (Nicholls and Perry, 2016), and sporting performance (Jowett and Cockerill, 2003). As such, enhancing our understanding of the coach–athlete relationship may have important implications for maximizing sporting and psychological outcomes among both players and coaches (Nicholls and Perry, 2016). There are three theoretical models that specifically attempted to conceptualize the coach–athlete relationship. These were proposed by Poczwardowski et al. (2002), LaVoi (2004), and Jowett (2007). Jowett created the 3+1 Cs model, which included complementarity, co-orientation, closeness, and commitment. Complementarity is the degree to which the behaviors of the athlete and coach relate to one another. Co-orientation represents the extent to which the athlete and coach have established common views on sporting and non-sporting matters. Closeness refers to the extent to which the athlete and coach care, support, and value each other. Finally, commitment relates to whether the athlete and coach intend to maintain their relationship. Jowett purported that the coach–athlete relationship is dynamic as both the coach and the athlete can influence the relationship and that it changes over time. LaVoi (2007) identified four main components in the coach–athlete relationship (e.g., authenticity, engagement, empowerment, and ability). Finally, Poczwardowski et al. (2002) and Poczwardowski (unpublished) conceptualized the coach–athlete relationship as recurring patterns of mutual care between coaches and athletes. At the present time, however, only Jowett developed a questionnaire to accompany her model (Jowett and Ntoumanis, 2004), whereas, Lavoi and Poczwardowski are yet to create a questionnaire. The questionnaire by Jowett and Ntoumanis is widely used across different populations and is a valid measure of this construct, so we felt it was appropriate her framework and questionnaire.

To our knowledge, scholars are yet to explore the extent to which the coach–athlete relationship changes over time. As such, the first purpose of this study was to address this gap in the literature and examine whether perceptions of the coach–athlete relationship changed over a period of 6 months. Although little is known about how the coach–athlete relationship may change over time, there is an association between this construct and achievement goals. Adie and Jowett (2010) examined the extent to which mastery-approach (i.e., striving to attain self-referenced competence), mastery-avoidance (i.e., avoiding self-referenced incompetence), performance-approach (i.e., striving to attain normative competence), and performance-avoidance goals (i.e., aiming to avoid normative incompetence; Elliot, 1999) were linked to athletes’ overall perception of the coach–athlete relationship. They revealed that athletes who perceived a closer and more committed relationship with their coach were more likely to adopt mastery-approach goals, but less likely to adopt mastery-avoidance goals. These findings were echoed by Isoard-Gautheur et al. (2016) who reported that a stronger perceived coach–athlete relationship was associated with mastery-approach goals. There is also evidence that links goals with how an athlete evaluates stress (Nicholls et al., 2014, 2016a). Athletes who adopt mastery-approach goals are more likely to view stressful situations as challenging, whereas athletes who endorse mastery-avoidance or performance-avoidance are more likely to experience threat when in stressful situations (Nicholls et al., 2014). Further, athletes who use goal re-engagement strategies are likely to experience challenge states, whereas goal disengagement strategies are more likely to generate threat appraisals (Nicholls et al., 2016a). Scholarly activity by Lochbaum and Smith (2015) revealed that mastery-approach goals are associated with superior performance in golf. As such, the coach–athlete relationship may be associated with sporting performance, via achievement goals.

Sport psychology researchers reported a link between the coach–athlete relationship and sporting performance. For example, Jowett and Cockerill (2003) interviewed 12 Olympic medalists regarding their experiences of the coach–athlete relationship. Findings revealed that the quality of the coach–athlete relationship was instrumental in helping the athletes perform well and thus win an Olympic medal. Other scholars examined this relationship via quantitative research designs. Mata and Da Silva Gomes (2013) examined the relationship between perceptions of coach–athlete relationship quality and goal achievement among two teams that won the most prestigious professional volleyball competitions (e.g., league and cup) and the four teams that made the league play offs, but failed to win. Volleyball players on the two winning teams perceived their coach’s leadership more favorably, were more satisfied with their coach, and perceived higher goal achievement than those on the four losing teams. Nikbin et al. (2014) examined perceptions of athletes’ commitment and trust toward their coach with performance among volleyball and futsal players from Iran. Both commitment to one’s coach and trust were significantly and positively associated with sporting performance. Vieira et al. (2015) explored perceptions of the coach–athlete relationship among medalist and non-medalist Under-18 volleyball players. The volleyball players who won a medal perceived that they were closer and more committed to their coaches than the non-medalists. It should be noted that the association between the coach–athlete relationship and sports performance is yet to be tested longitudinally. Assessing this relationship longitudinally will allow scholars to assess the predictive powers of the coach–athlete relationship. The second purpose of this study was to assess the relationship between the coach–athlete relationship and goal achievement.

The aim of this study was to quantitatively assess some of the theoretical and empirical assertions made by Jowett (2007). Firstly, we assessed whether the coach–athlete relationship changed over six months, from Time 1 (T1) to Time 2 (T2). Based on Jowett’s (2007) empirical model, we predicted that the coach–athlete relationship would change from T1 to T2. We also examined whether the coach–athlete relationship was associated with goal achievement at the initial measurement and whether perception of the coach–athlete relationship predicted goal achievement six months later. In accordance with existing research (e.g., Jowett and Cockerill, 2003; Nikbin et al., 2014; Vieira et al., 2015), we predicted that the coach–athlete relationship would be associated with goal achievement and that it would also positively predict goal achievement six months later.

## Materials and Methods

### Participants

One-hundred and four male F. A. Premier League academy soccer players, aged between 9 and 20 years old (*M*_age_ = 14.19, *SD* = 3.56) participated in this study. Participants reported playing academy football for between 0 and 12 years (*M*_years_ = 3.61, *SD* = 2.74). The participants were White British (*n* = 96), Black British – African (*n* = 2), Black British –Caribbean (*n* = 1), Mixed Black Caribbean and White (*n* = 1), Other Mixed (*n* = 1), White Irish (*n* = 1), Other white (*n* = 1), or Mixed Asian and White (*n* = 1).

### Measures

#### Coach–Athlete Relationship

We used the Coach Athlete Relationship Questionnaire (CART-Q; Jowett and Ntoumanis, 2004) to assess the players’ perceptions of the coach–athlete relationship. The CART-Q is an 11-item questionnaire that measures closeness, commitment, and complementarity. The scale includes questions such as “I trust my coach,” “I am committed to my coach,” and “When I am coached by my coach, I adopt a friendly stance.” The questions were answered on a seven-point Likert-type scale, which ranged from 1 = *strongly disagree* to 7 = *strongly agree*. Other scholars reported satisfactory psychometric properties for this measure including construct and factorial validity, criterion validity, and internal consistency (e.g., Jowett and Ntoumanis, 2004; Olympiou et al., 2008; Jowett, 2009). Moreover, previous studies also provided support for the use of an overall coach–athlete relationship score and used this measure among similar samples to the present study (e.g., Jowett, 2008).

#### Goal Achievement

The 12-item Attainment of Sport Achievement Goal Scale (A-SAGS; Amiot et al., 2004) assessed the extent to which athletes believed they had achieved mastery (e.g., “Performed my football skills correctly”), self-referenced goal achievement (e.g., “Did my best performance of the season”), and normative goal achievement (e.g., “Outplayed other footballers”). Participants answered the questions on a seven-point Likert-type scale, which was anchored at 1 = *‘not at all’* and 7 = *‘strongly.’* Previous studies reported satisfactory psychometric properties for this measure (e.g., Amiot et al., 2004; Gaudreau and Antl, 2008; Nicolas et al., 2011). Moreover, these studies provided support for the use of an overall score and used this measure with similar samples to those in the present study (e.g., Nicolas et al., 2011).

### Procedure

We obtained ethical approval from a university’s departmental ethics committee. Following approval, we purposively sampled F. A. Premier League academy players within one academy by distributing information letters, consent forms, and assent forms to all players within the academy, with the aim of recruiting as many players as possible. We obtained informed consent from all participants aged 18 years and over, informed assent from players aged 17 years and below, and parental consent from all players who were aged 17 years and below. Participants received a questionnaire pack containing demographic information, the CART-Q (Jowett and Ntoumanis, 2004) and the A-SAGS (Amiot et al., 2004) during November (T1) and then six months later in May (T2). Participants were instructed to complete the CART-Q in regards to one of their lead coaches. There are two lead coaches in the Foundation phase, two in the youth development phases, and five coaches in the professional development phase. We did not ask participants to identify the coach the completed the questionnaire about, because we thought that participants might less inclined to provide honest answers, but the players were instructed to complete the questionnaire in regards to the same coach at T1 and T2. In total, 104 players completed T1 and 52 players completed both T1 and T2 assessments. Of the 104 players who completed the assessments at T1, 35 players were released, 12 players were injured, five players were on loan at another club, and two players had joined another club when the T2 assessments occurred. All of the players absent from the academy during T2 assessments were sent questionnaires to their home address in stamped address envelope, but only two players returned the questionnaires. Other than these two players, all participants completed the questionnaires in the presence of Keith Earle, who is a Health and Care Professions Council Registered Psychologist. Keith Earle was present to answer any questions the athletes had and to clarify the meaning of the questions if the players struggled to comprehend them.

### Data Screening

Firstly, we inspected the data for missing values. As very few item responses were missing (*i* = 12), missing responses were replaced with the mean of the item responses of the corresponding scale (ipsatised item replacement; Graham et al., 2003). We then computed Cronbach’s alphas for our variables which were all satisfactory (see **Table 1**; >0.70; Nunnally and Bernstein, 1994). Finally, following recommendations by Tabachnick and Fidell (2007), we screened data for multivariate outliers. No participant showed a Mahalanobis distance larger than the critical value of χ^2^(16) = 39.25, *p* < 0.001, therefore, all data were retained for further analyses. As previous research by Jowett (2008) did not find that age was a significant moderator between the coach–athlete relationship and self-concept among academy players of a similar age, we did not analyze the data based on age group categories.

**Table 1:** Descriptive statistics, Cronbach’s alphas, and bivariate correlations.

Variable	1	2	3	4	5	6	7	8	9	10	11	12	13	14	15	16
**Time 1**																
(1) Coach–athlete																
(2) Closeness	0.95^∗∗∗^															
(3) Commitment	0.89^∗∗∗^	0.81^∗∗∗^														
(4) Complementarity	0.90^∗∗∗^	0.79^∗∗∗^	0.67^∗∗∗^													
(5) Achievement	0.19	0.14	0.17	0.22^∗^												
(6) Mastery achievement	0.33^∗∗^	0.24^∗^	0.27^∗∗^	0.39^∗∗∗^	0.69^∗∗∗^											
(7) Self-referenced achievement	0.16	0.11	0.19	0.15	0.88^∗∗∗^	0.42^∗∗∗^										
(8) Normative achievement	0.08	0.06	0.03	0.12	0.89^∗∗∗^	0.57^∗∗∗^	0.62^∗∗∗^									
**Time 2**																
(9) Coach–athlete	0.79^∗∗∗^	0.73^∗∗∗^	0.72^∗∗∗^	0.63^∗∗∗^	0.21	0.40^∗∗^	0.15	0.10								
(10) Closeness	0.71^∗∗∗^	0.71^∗∗∗^	0.62^∗∗∗^	0.55^∗∗∗^	0.22	0.42^∗∗^	0.15	0.10	0.93^∗∗∗^							
(11) Commitment	0.73^∗∗∗^	0.69^∗∗∗^	0.73^∗∗∗^	0.51^∗∗∗^	0.18	0.33^∗^	0.16	0.06	0.93^∗∗∗^	0.78^∗∗∗^						
(12) Complementarity	0.75^∗∗∗^	0.62^∗∗∗^	0.65^∗∗∗^	0.70^∗∗∗^	0.18	0.36^∗∗^	0.10	0.11	0.93^∗∗∗^	0.77^∗∗∗^	0.81^∗∗∗^					
(13) Achievement	0.26	0.23	0.19	0.25	0.65^∗∗∗^	0.39^∗∗^	0.59^∗∗∗^	0.61^∗∗∗^	0.40^∗∗^	0.35^∗^	0.38^∗∗^	0.38^∗∗^				
(14) Mastery achievement	0.48^∗∗∗^	0.41^∗∗^	0.42^∗∗^	0.44^∗∗^	0.51^∗∗∗^	0.53^∗∗^	0.39^∗∗^	0.45^∗∗^	0.58^∗∗∗^	0.55^∗∗∗^	0.52^∗∗∗^	0.53^∗∗∗^	0.80^∗∗∗^			
(15) Self-referenced achievement	0.19	0.20	0.11	0.19	0.51^∗∗∗^	0.18	0.55^∗∗∗^	0.44^∗∗^	0.35^∗^	0.31^∗^	0.35^∗^	0.31^∗^	0.91^∗∗∗^	0.61^∗∗∗^		
(16) Normative	0.09	0.07	0.04	0.12	0.69^∗∗∗^	0.40^∗∗^	0.57^∗∗∗^	0.71^∗∗∗^	0.19	0.13	0.17	0.22	0.90^∗∗∗^	0.62^∗∗∗^	0.71^∗∗∗^	
*M*	67.05	25.48	17.31	24.26	63.89	23.48	19.90	20.51	66.04	24.85	17.42	23.77	62.98	22.53	20.08	20.37
*SD*	8.42	3.35	2.79	3.06	9.91	2.23	5.01	4.45	7.41	2.87	2.44	2.70	9.90	6.69	4.64	3.90
Cronbach’s alpha	0.93	0.90	0.78	0.76	0.90	0.73	0.89	0.91	0.93	0.90	0.80	0.79	0.93	0.73	0.93	0.91

*N = 104 for Time 1; *N* = 52 for Time 2. Time 2 = six months after Time 1.*

*^∗^*p* < 0.05; ^∗∗^*p* < 0.01; ^∗∗∗^*p* < 0.001.*

### Data Analysis

To examine the associations between the coach–athlete relationship and goal achievement, we firstly examined bivariate (Pearson) correlations between all variables. This also allowed us to investigate the stability of the coach–athlete relationship by examining the correlations between the coach–athlete relationship at T1 and T2. Next, we conducted a series of multiple regression analysis to investigate the longitudinal relationship between the coach–athlete relationship and goal achievement. Goal achievement from T1 was entered at Step 1, to control for baseline levels of goal achievement. The coach–athlete relationship from T1 was then entered at Step 2 (for which we used the composite score which is reflective of the overall coach–athlete relationship). This analysis was repeated for overall goal achievement and the three subscales of goal achievement (i.e., mastery, self-referenced, and normative).

## Results

### Bivariate Correlations

We inspected the bivariate correlations between all variables (see **Table 1**). As expected, the subscales of both the coach–athlete relationship and goal achievement showed strong inter-correlations within waves. Moreover, **Table 1** shows that the coach–athlete relationship remained relatively stable between T1 and T2, as indicated by T1-T2 correlations of 0.79 (for overall score), 0.71 (for closeness), 0.73 (for commitment), and 0.70 (for complementarity). Finally, the results show the coach–athlete relationship (overall score and all sub-scale scores) was associated with achievement mastery goals, both cross-sectionally (0.33) and longitudinally (0.48). The coach–athlete relationship, however, did not correlate with self-referenced or normative goal achievement.

### Multiple Regression Analyses

We then conducted a series of multiple regression analyses (see **Table 2**). Results showed that the coach–athlete relationship predicted residual increases in the achievement of mastery goals over time.^1^

**Table 2:** Summary of regression analyses predicting goal achievement at T2.

	Goal achievement T2		Mastery T2		Self-referenced T2		Normative T2	
Predictors at T1	Δ*R*^2^	*B*	Δ*R*^2^	*B*	Δ*R*^2^	*B*	Δ*R*^2^	*B*
Step 1	0.421^∗∗∗^		0.285^∗∗∗^		0.297^∗∗∗^		0.507^∗∗∗^	
DV		0.65^∗∗∗^		0.53^∗∗∗^		0.55^∗∗∗^		0.71^∗∗∗^
Step 2	0.011		0.059^∗^		0.010		0.001	
DV		0.62^∗∗∗^		0.39^∗∗^		0.53^∗∗∗^		0.71^∗∗∗^
Coach–athlete relationship		0.11		0.28^∗^		0.10		0.03

*N = 52. Cronbach’s alphas for the data from participants who participated at both time points (*N* = 52) exceeded 0.70 for all scales at both time points. T2 = six months after T1. DV = dependent variable at T1.*

*^∗^*p* < 0.05; ^∗∗^*p* < 0.01; ^∗∗∗^*p* < 0.001.*

## Discussion

The aim of this paper was to explore whether perceptions of the coach–athlete relationship changed over six months and if the coach–athlete relationship predicted goal achievement among F. A. Premier League academy soccer players. Our prediction that the coach–athlete relationship would change across the six months was not supported. The players perceived that the quality of their coach–athlete relationship remained relatively stable. Our second prediction that the coach–athlete relationship would be associated with achievement goals was partially supported, as mastery goal achievement was positively associated with the coach–athlete relationship at T1 and T2. Neither self-referenced goal achievement nor normative goal achievement, however, correlated with the coach–athlete relationship at T1 or T2. Our final prediction that the players’ perceptions of their coach–athlete relationship would predict goal achievement was also partially supported. Players’ perceptions of their coach–athlete relationship positively predicted mastery goal achievement, but not self-reference goal achievement or normative goal achievement.

Jowett (2007) proposed that the coach–athlete relationship changes over time, which was not supported in the present study. Indeed, perceptions of the coach–athlete relationship remained relatively stable over six months among the participants. It is feasible that examining the coach–athlete relationship over six months is not long enough to observe changes in this construct. A challenge of adopting this approach, particularly among developmental athletes in team sports is that these players tend to have a new coach every year so only ever spend 11 months with the same coach. It is also plausible; however, that the coach–athlete relationships were already formed when we collected the data, so it would be interesting to track perceptions of the coach–athlete relationship from the first coaching session an athlete has with his or her coach and monitor this relationship over a prolonged period of time as this is when changes may occur. From a practical point of view, we were given a period of six months to conduct the study, so were limited by the club. This is a challenge of conducting research within professional sports settings. In her model, Jowett proposed that the coach–athlete relationship changes over time, but provided little information on the time required to see such changes, and we believed that assessing this relationship over six months would be sufficient. It is clear that scholars may need to assess this relationship over longer periods or more frequently. We did not assess whether previously reported stressors among academy players influenced perceptions of the coach–athlete relationship. It would be interesting to monitor perceptions of the coach–athlete relationship throughout contractual negotiations, after de-selection, and after the outcome of crucial matches or competitions, as Reeves et al. (2009) found that these were stressful incidents among another sample of F. A. Premier League academy players. This would require players to complete the CART-Q (Jowett and Ntoumanis, 2004) on a regular basis throughout the season after being de-selected, during contractual negations, or after winning or losing matches. These stressors may influence players’ perceptions of the coach–athlete relationship, as recent research found an association between stress appraisals and the coach–athlete relationship (Nicholls et al., 2016b). It should be noted, however, that players in F. A. Premier League academies are required to complete a variety of different questionnaires on a weekly basis as a part of EPPP regulations, so future research with this population should not be too time consuming for the players.

We found that the athlete’s perception of the coach–athlete relationship at T1 predicted mastery goal achievement at T2. That is, the academy players who perceived a stronger relationship with their coach were more likely to report higher levels of mastery goal achievement six months later. We offer an explanation for this finding. The academy players who rated their coach–athlete relationship highly may have deployed a mastery-goal approach in the six months preceding T2. Both Adie and Jowett (2010) and Isoard-Gautheur et al. (2016) reported that a stronger perceived coach–athlete relationship was associated with mastery-approach goals. Furthermore, scholarly activity by Lochbaum and Smith (2015) revealed that mastery-approach goals are associated with superior performance in golf. As such, the academy players in the present study might have deployed more mastery-approach goals, which subsequently aided their goal achievement. Prospective research is required to test the efficacy of this explanation and thus explore whether athletes who perceive a stronger relationship with their coach deploy more mastery-approach goals and thus perform better.

From an applied perspective, our findings highlight the potential importance of the coach–athlete relationship, among developmental athletes in elite team sport settings. Although there are established guidelines for developing the coach–athlete relationship (e.g., Mageau and Vallerand, 2003; Rhind and Jowett, 2010), the effects of these recommendations on sports performance are currently unknown. Our findings suggest that enhancing the coach–athlete relationship could help athletes achieve their goals more effectively and thus raise performance. Research is required, however, to test this assertion. Applied sport psychology practitioners could dedicate some of their time to helping coaches forge strong relationships with their players in an attempt to enhance commitment, closeness, and complementarity among players, given the positive association with mastery achievement goals.

A limitation of this study is that we did not record the duration of the coach–athlete relationships, because each age group has different coaches, so players generally spend only 1 year with each coach, other than the older players within the academy. This is a limitation, because Jowett (2008) revealed that the length of the coach–athlete relationship impacted upon perceived relationship quality among academy athletes. Another limitation of this study relates to the sample. Our sample is relatively homogenous in that it comprised of elite male team sport athletes. Other research could test the generalisability of these findings among female and individual sport athletes. There was also a high dropout rate from T1 to T2. Thus, the study may have lacked statistical power to detect smaller effects (Cohen, 1992). This, however, is consistent with other longitudinal research in an elite F. A. Premier League environment (e.g., Reeves et al., 2011b). Some players were released between T1 and T2, injured during the T2 collection period, or moved to another club, which meant they were not available to complete the T2 assessment and did not return the mailed questionnaires. A challenge of collecting longitudinal data in elite environments is the availability of players to provide data across all time points (see Nicholls et al., 2006, 2009a,b; Reeves et al., 2011b). Finally, whereas previous research (e.g., Jowett, 2008) suggested that age does not moderate coach–athlete relationship, future research is required to explore this further in samples with larger age ranges than the present study.

## Conclusion

We found that the coach–athlete relationship remained relatively stable across two time points, which were six months apart. In order to cement our understanding of the coach–athlete relationship, it would be interesting to explore fluctuations over multiple time points and take into consideration factors that influence the coach–athlete relationship such as stressors. The coach–athlete relationship appears to be an important predictor of mastery goal achievement, so performance in soccer academies could be maximized by incorporating coach–athlete relationship training in coach education programs.

## Ethics Statement

This study was approved by the School of Life Sciences’ ethics committee. All participants provided written consent to take part in this research.

## Author Contributions

All authors listed, have made substantial, direct and intellectual contribution to the work, and approved it for publication.

## Conflict of Interest Statement

The authors declare that the research was conducted in the absence of any commercial or financial relationships that could be construed as a potential conflict of interest. The reviewer IS and handling Editor declared their shared affiliation, and the handling Editor states that the process nevertheless met the standards of a fair and objective review.

## Footnotes

1 We ran an additional logistic regression investigating if the coach–athlete relationship predicted dropout (as a binary variable). The coach–athlete relationship emerged as a non-significant predictor (*p* > 0.05).
